# Non-invasive pulse arrival time is associated with cardiac index in pediatric heart transplant patients with normal ejection fraction

**DOI:** 10.1088/1361-6579/ad61b9

**Published:** 2024-07-22

**Authors:** Soon Bin Kwon, Bennett Weinerman, Daniel Nametz, Murad Megjhani, Isaac Lee, Anthony Habib, Oliver Barry, Soojin Park

**Affiliations:** 1 Department of Neurology, Columbia University Vagelos College of Physicians and Surgeons, New York, NY, United States of America; 2 Program in Hospital and Intensive Care Informatics, Department of Neurology, Columbia University Vagelos College of Physicians and Surgeons, New York, NY, United States of America; 3 Columbia University College of Physicians and Surgeons, Division of Pediatric Critical Care and Hospital Medicine, New York, NY, United States of America; 4 Columbia University College of Physicians and Surgeons, Division of Pediatric Anesthesiology, New York, NY, United States of America; 5 Columbia University College of Physicians and Surgeons, Division of Pediatric Cardiology, New York, NY, United States of America; 6 NewYork-Presbyterian Hospital, Columbia University Irving Medical Center, New York, NY, United States of America; 7 Department of Biomedical Informatics, Columbia University, New York, NY, United States of America

**Keywords:** cardiac index, pulse arrival time, non-invasive, heart rate, ejection fraction, pediatric, cardiac output

## Abstract

*Objective.* Cardiac Index (CI) is a key physiologic parameter to ensure end organ perfusion in the pediatric intensive care unit (PICU). Determination of CI requires invasive cardiac measurements and is not routinely done at the PICU bedside. To date, there is no gold standard non-invasive means to determine CI. This study aims to use a novel non-invasive methodology, based on routine continuous physiologic data, called Pulse Arrival Time (PAT) as a surrogate for CI in patients with normal Ejection Fraction (EF). *Approach.* Electrocardiogram (ECG) and photoplethysmogram (PPG) signals were collected from beside monitors at a sampling frequency of 250 samples per second. Continuous PAT, derived from the ECG and PPG waveforms was averaged per patient. Pearson’s correlation coefficient was calculated between PAT and CI, PAT and heart rate (HR), and PAT and EF. *Main Results.* Twenty patients underwent right heart cardiac catheterization. The mean age of patients was 11.7 ± 5.4 years old, ranging from 11 months old to 19 years old, the median age was 13.4 years old. HR in this cohort was 93.8 ± 17.0 beats per minute. The average EF was 54.4 ± 9.6%. The average CI was 3.51 ± 0.72 l min^−1^ m^−2^, with ranging from 2.6 to 4.77 l min^−1^ m^−2^. The average PAT was 0.31 ± 0.12 s. Pearson correlation analysis showed a positive correlation between PAT and CI (0.57, *p* < 0.01). Pearson correlation between HR and CI, and correlation between EF and CI was 0.22 (*p* = 0.35) and 0.03 (*p* = 0.23) respectively. The correlation between PAT, when indexed by HR (i.e. PAT × HR), and CI minimally improved to 0.58 (*p* < 0.01). *Significance.* This pilot study demonstrates that PAT may serve as a valuable surrogate marker for CI at the bedside, as a non-invasive and continuous modality in the PICU. The use of PAT in clinical practice remains to be thoroughly investigated.

## 1. Introduction

In the pediatric intensive care unit (PICU), patients are at high risk for alterations in their physiology, leading to hemodynamic instability. In the context of fluctuating hemodynamics, a goal of management is adequate end organ perfusion and oxygen delivery (Massey *et al* 2023). In both adults and pediatrics, cardiac output (CO) is a key factor to assess cardiac function and organ perfusion. In pediatrics, due to the variable patient size, CO is conventionally indexed by body surface area (BSA) as cardiac index (CI).

CI serves as an important indicator of cardiovascular status. The gold standard for calculating CI is via invasive cardiac catheterization, which is not routinely performed in the PICU. Using cardiac catheterization, CI is directly calculated via two methods, the modified Fick equation and/or thermodilution. In adults, pulmonary artery catheterization is done more routinely at the bedside; however, in pediatrics, pulmonary artery catheterization is rarely done outside of the cardiac catheterization laboratory.

Several technologies provide other means to approximate or assess pediatric CI (Maslow 2017) such as bedside Pulse index Contour Cardiac Output (PiCCO), and impedance cardiography (Tirotta *et al* 2017). PiCCO provides an excellent assessment for continuous CI, but requires placement of invasive venous catheters (Aslan *et al* 2020). Trademarked brands, such as VISMO®, provide estimated continuous CO (esCCO) based on ECG, pulse-oximeter wave, and cuff arterial BP; however, acceptable performance for clinical application is questionable (Terada *et al* 2014, Anand *et al* 2021, Fot *et al* 2023). Impedance cardiography has gained attraction as a non-invasive modality to measure CI; however, this methodology requires additional electrodes, and the measured signals can be disrupted by respirations and patient movement (Nederend *et al* 2017). Novel applications of impedance cardiography and carotid waveform analytics have been associated with cardiac efficiency and left ventricular function, though largely in adult models (Cooper *et al* 2021, Kuang *et al* 2021, Cheng *et al* 2023, Suriani *et al* 2023).

Another commonly used, non-invasive modality to evaluate cardiac health is echocardiography, where ejection fraction (EF), is used as a quantitative measure of systolic function. EF is an assessment with limitations (Dittoe *et al* 2007), but widely used to stratify systolic ventricular dysfunction (Ziaeian and Fonarow 2016, Heidenreich *et al* 2022). EF depicts an incomplete picture of a patient’s overall cardiovascular health (Shakir and Rasul 2009), but has been demonstrated to be associated with morbidity and mortality (Sevilla Berrios *et al* 2014). An additional limitation is that echocardiography offers only a snapshot of information (Thavendiranathan *et al* 2013), which limits clinical utility in patients with rapidly changing physiology (Elhoff *et al* 2016, Monnet and Teboul 2017); however, newer imaging modalities are evolving in attempts to capture the complex interplay between respiratory and ventricular physiology (Friedberg 2018).

**Figure 1.**
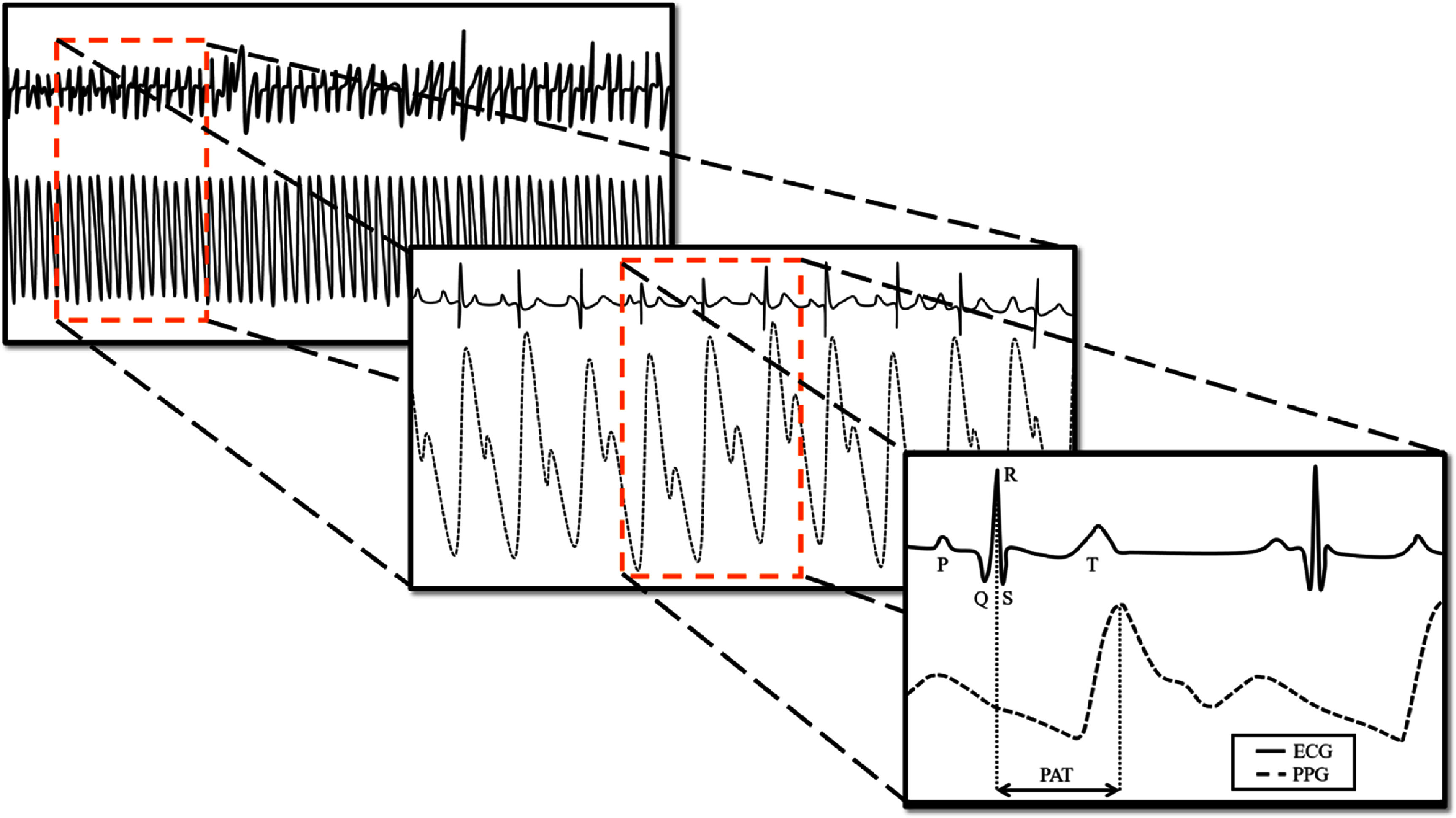
Schematic of Pulse Arrival Time (PAT) calculated as the time delay between electrocardiography (ECG) R-peak and the photoplethysmography (PPG) peak.

Other continuous means of estimating CI and measures of effective cardiac function at the patient bedside include cerebral/somatic near infrared spectroscopy (Hansen *et al* 2022), central venous pressure (Liu *et al* 2016), as well as physiologic markers such as heart rate (HR), mean arterial pressure, capillary refill, urine output, and arterial lactate levels (Lazzeri *et al* 2015, Shahsavarinia *et al* 2020, Patel *et al* 2022, Yu *et al* 2022). These routine monitoring systems and values to assess CI do not independently have a strong correlation with a patient’s CI or hemodynamic status (Marik *et al* 2008, Sevransky 2009, Bhalala *et al* 2012, Bakker 2016, Engoren *et al* 2017). In the pursuit of a non-invasive, continuous measurement of CI for critically ill pediatric patients, we examined a waveform derived value called Pulse Arrival Time (PAT) in relation to patient CI values obtained during cardiac catheterization. PAT is defined as the time delay between the electrical depolarization of the heart’s left ventricle, measured by electrocardiogram (ECG), and the peak point in the associated waveform in the patient’s photoplethysmogram (PPG) (figure 1). There are many ways to derive PAT, none of which have been rigorously studied in the pediatric population. Thus, we first chose a routinely cited methodology that defines PAT from ECG R-peak to PPG peak. The process of R-peak to PPG peak takes six steps of transformation starting from electric excitation of the ventricle in the form of the R peak of the ECG, translated to cardiac contraction, generation of pressure impulse, propagation of the pressure wave, displacement of intravascular blood volume and finally alteration in light intensity as detected by the pulse oximeter. Each of these steps has different transfer functions, which are affected by autonomic activities, respiration, blood pressure and disease pathophysiology (Yuda *et al* 2020). Within the PAT waveform analysis there is the potential for robust physiologic relationships, which could provide more clinically useful information than HR and EF alone. We hypothesized that PAT would be influenced by either ventricular function, HR or both, making it a potential surrogate for estimating CI in pediatric patients.

In adults, PAT has been shown to be inversely correlated to systolic blood pressure; however, the exact relationship appears to be patient specific and variable across subjects (Lee *et al* 2019, Finnegan *et al* 2021, Heimark *et al* 2022). None of these studies evaluate relationships between PAT and CI, nor did they focus on any pediatric cohorts.

Knowledge of the CI of a patient at the bedside with a non-invasive and continuous monitoring modality would greatly enhance the management of critically ill pediatric patients. This retrospective observational cohort study investigated the relationship between PAT and cardiac catheterization derived CI in patients, while also determining the influence of HR and EF on the metric.

## 2. Methods

We performed a retrospective observational cohort study at a single academic institution with a dedicated pediatric heart failure and pediatric heart transplant department. Patients who were undergoing non-urgent right heart catheterization after orthotopic heart transplant (OHT) were included. Patients who did not have biventricular anatomy or who were on ventricular assist devices were excluded. We chose post-OHT patients since they routinely undergo right heart catheterization for evaluation of their transplanted organ. These patients also have structurally normal cardiac anatomy, potentially making their physiologic findings more applicable to the PICU patient population. While this patient population is generally well, a subset may be referred for right heart catheterization if there are concerns for decreased cardiac function and/or signs of organ rejection.

Right heart catheterization was performed by an interventional cardiologist through the femoral or internal jugular vein, while the patient was under general anesthesia performed by a pediatric anesthesiologist. Patients were endotracheally intubated, assisted with a laryngeal mask airway (LMA) or sedated with spontaneous breathing and end tidal carbon dioxide monitoring at the anesthesiologist’s discretion. Data was collected for analysis from the start of the pediatric cardiac catheterization. The Columbia University IRB committee approved this research study (Protocol # AAAU5398). Research was conducted in accordance with the principles embodied in the Declaration of Helsinki and accordance with local statutory requirements. This study was a retrospective, non-treatment, observational review of physiologic waveform data that was already collected as part of routine standard of care, thus informed consent was waived. Echocardiographic measurement of EF was performed by a certified pediatric echocardiographer at the discretion of the pediatric heart failure team. EF was calculated using Biplane Simpson’s method (Dittoe *et al* 2007).

### 2.1. Study population

Twenty patients with history of OHT undergoing routine cardiac catheterization were included in the study. Demographic data including age and gender were collected. Clinical data were collected including height, weight, BSA, time between OHT to recorded cardiac catheterization, reason for transplant, time from cardiac catheterization to echocardiogram and transthoracic echocardiographic measurements (after cardiac catheterization) (table 1). The study included 7 female and 13 male patients. For all patients, gender was congruent with the patient’s sex, based on parental or patient preferences where appropriate. The patients’ ages ranged from 11 months to 19 years old with an average age of 11.7 years. The time from heart transplant to cardiac catheterization, where data was collected, ranged from 23 d to 192.9 months, with patients on average being 71.6 months from their heart transplant surgery. The average age of the patient when they received their OHT was 75.6 months, with a range of 1.3 months to 17 years old.

**Table 1.** Demographic data for post-OHT patients undergoing right heart cardiac catheterization.

	Mean (STD)
Age (Years)	11.7 (5.4)
Height (cm)	144.1 (30.1)
Weight (kg)	46.4 (23)
Body Surface Area (m^2^)	5.7 (19.6)
Time from Transplant (Months)	71.6 (66.9)
Age at time of Transplant (Months)	75.6 (66.5)
Gender (F:M)	7:13
Intubated (Y:N)	3:17

CO was calculated using mixed venous saturation, systemic saturation and measured hemoglobin content. Oxygen consumption (VO_2_) was estimated based on calculations as described by Seckeler *et al* (2015). CO was then indexed by each patient’s BSA to obtain CI. All calculations were performed when the patient was breathing 21% oxygen. Five 3 M Red Dot ECG electrodes (St. Paul, Minnesota) were placed in usual fashion and a Masimo RD Set Neo Pulse Oximeter (Irvine, California) was placed on the index finger. Digital physiologic data (ECG and PPG) were collected from Philips Intellivue monitors (Amsterdam, Netherlands) at a sampling frequency of 250 samples per second using ICM Plus software (Cambridge Enterprise, United Kingdom). Physiologic data periods used for analysis were from 20 March 2023 to 5 June 2023.

### 2.2. Statistical analysis

All data analyses were performed using MATLAB 2020a (MathWorks, Massachusetts). ECG waveform was first observed manually to check for the validity of the measurement and artifact waveforms were removed. Selected ECG waveforms and their corresponding PPGs were used for further processing. ECG R-peaks were obtained using Pan and Tompkins algorithm (Pan and Tompkins 1985). The time between R-peak and peak of PPG was calculated as PAT (figure 1). Additionally, we calculated PAT—Foot (PAT_Foot_), derived as the time difference from R-peak of the QRS complex of the ECG to the base/foot of the PPG waveform. We also calculated PAT—Qwave (PAT_Q_) as the time difference between the Q-wave in the QRS complex of the ECG to the peak of PPG waveform (supplemental figure 1).

After calculating PAT for all selected waveforms, PAT, PAT_Foot_, and PAT_Q_ were averaged per patient. Pearson’s correlation coefficient was calculated between PAT and CI, PAT_Foot_ and CI, PAT_Q_ and CI, PAT and HR, and PAT and EF during the same time points where ECG was selected. PAT was also indexed to HR, as PAT multiplied by HR. The correlation between the indexed PAT and CI was then calculated. We also indexed PAT by the Cardiac Ejection Time (PAT_ET_). For each patient we calculated individual ejection time as the duration of each PPG waveform (supplemental figure 1). For each patient, individual PAT values were divided by the subsequent Ejection Time. The average PAT_ET_ value was then calculated per patient and correlated with CI. As EF is often used as a metric for stroke volume, and CO is defined as the product of stroke volume and HR, we calculated the Pearson’s correlation between the product of EF and HR and CI.

## 3. Results

Demographics of patients are shown in table 1. The most common indication for OHT was dilated cardiomyopathy (50%). The average time from cardiac catheterization to echocardiogram was 5.6 d. Three of the twenty (15%) patients had an endotracheal tube placed during the cardiac catheterization, eleven had an LMA in place (55%) and the remaining seven (35%) patients had a nasal cannula with end tidal carbon dioxide monitoring.

**Figure 2.**
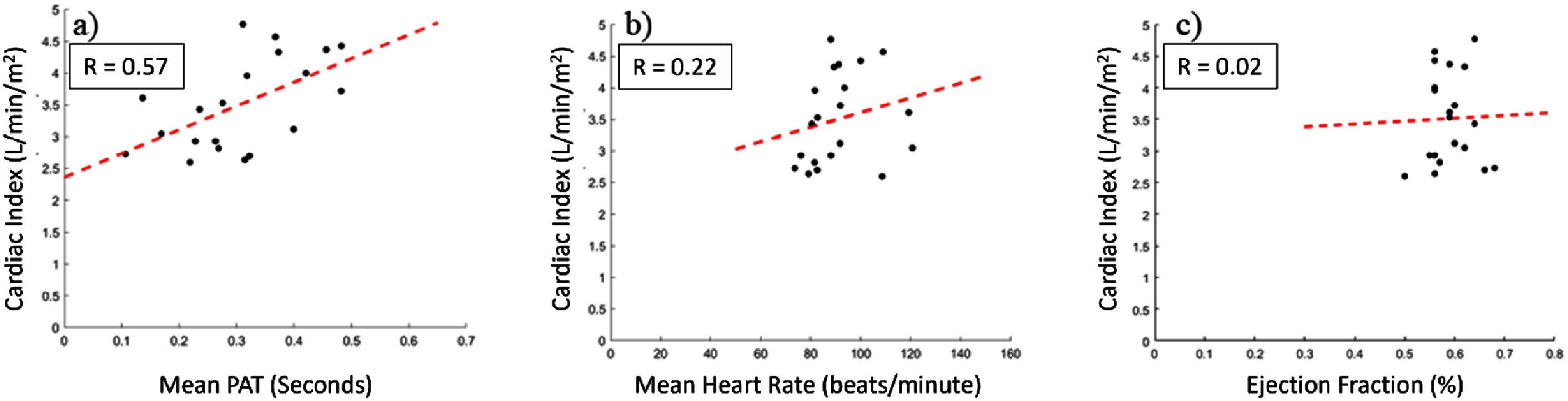
Pearson’s correlation between (a) Cardiac Index (CI) and Pulse Arrival Time (PAT), (b) CI and average heart rate, and (c) CI and Ejection Fraction.

The duration of cardiac catheterization data collection averaged 23 min. The average HR in this cohort was 94.0 ± 17.2 beats per minute. The average EF was 54.4 ± 9.6%, which ranged from 50 to 68%. The average CI was 3.51 ± 0.72 l min^−1^ m^−2^, with a range spanning 2.6 to 4.77 l min^−1^ m^−2^. The average PAT was 0.31 ± 0.12 s. Pearson correlation analysis showed a positive correlation between CI and PAT (0.57, *p* < 0.01). Pearson correlation between CI vs HR and CI vs EF was 0.22 (*p* = 0.35) and 0.03 (*p* = 0.23) respectively (figure 2). The correlation between PAT_Foot_ and CI was 0.30 (*p* = 0.20) (supplemental figure 2). The correlation between PAT_Q_ and CI was −0.21 (*p* = 0.34) (supplemental figure 3). Additionally, the correlation between HR and PAT, as well as EF and PAT were −0.06 (*p* = 0.82) and −0.18 (*p* = 0.46) respectively (table 2).

**Table 2.** Average Heart Rate (HR), Ejection Fraction and Pulse Arrival Time (PAT). Also shown is PAT indexed by HR (PAT x HR), PAT indexed by Ejection Time (PAT_ET_), PAT calculated from the Q-wave of the ECG complex to the plethysmogram peak (PAT_Q_), and PAT calculated from the R-peak of the QRS complex to the foot of the plethysmogram waveform. These permutations of PAT were then correlated with Cardiac Index.

	Mean (STD)		Correlation with CI (*p* value)		Correlation with PAT (*p* value)	
Heart Rate (beat/min)	94.0	(17.2)	0.22	(0.35)	−0.06	(0.82)
Ejection Fraction (Percentage)	54.4	(9.6)	0.03	(0.23)	−0.18	(0.46)
PAT (Seconds)	0.31	(0.12)	0.57	(<0.01)		
PAT × HR	7333.3	(2801.4)	0.58	(<0.01)		
PAT_ET_	0.54	(0.18)	0.37	(0.11)		
PAT_Foot_	0.23	(0.09)	0.30	(0.20)		
PAT_Q_	0.0089	(0.0056)	−0.21	(0.34)		
EF × HR	54.35	(9.9)	0.29	(0.23)		
Mean Measurement time (Minutes)	23.4	(13.4)				
Cardiac Index (l min^−1^ m^−2^)	3.5	(0.7)				

**Figure 3.**
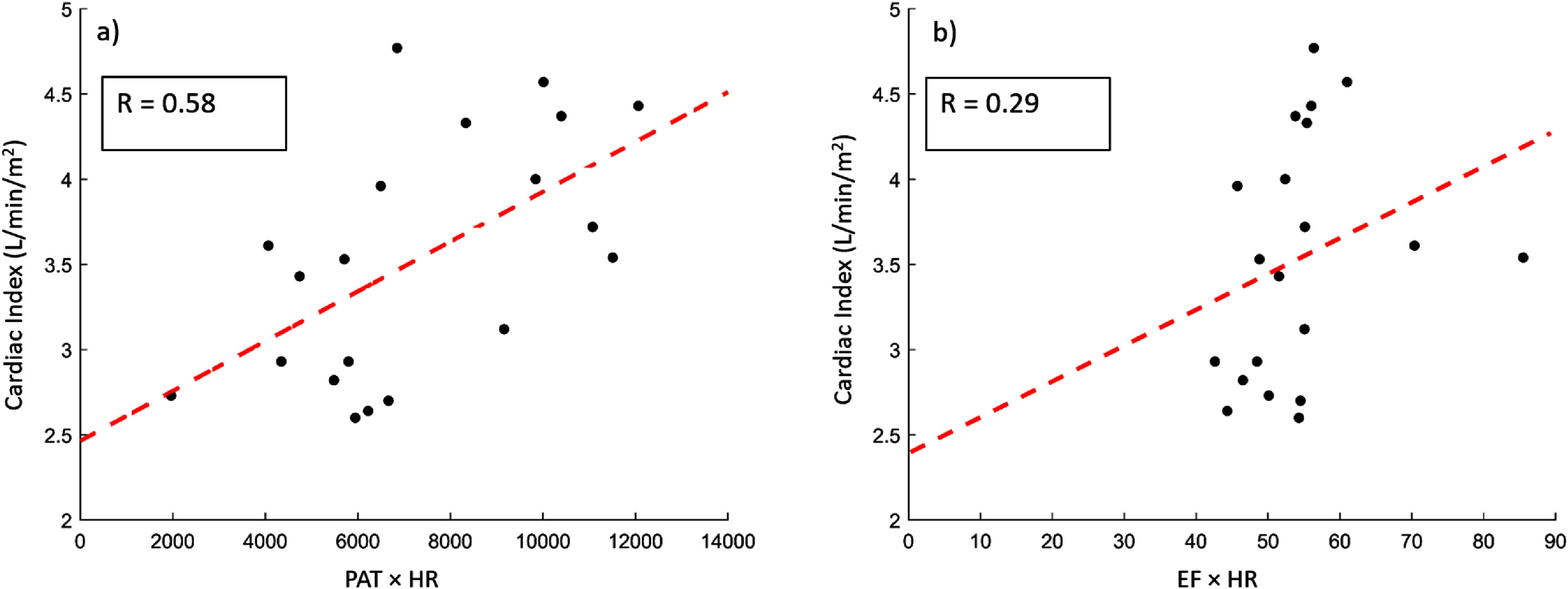
Pearson’s correlation between Pulse Arrival Time (PAT), indexed by heart rate (HR), and Cardiac Index (CI) as well as the correlation between Ejection Fraction (EF) indexed by HR and CI. (a) PAT multiplied by HR, (b) EF multiplied by HR.

We attempted to normalize PAT to HR, and correlate the findings to CI. The correlation between PAT multiplied by HR (i.e. PAT × HR) and CI was 0.58 (*p* < 0.01). We also tried to normalize PAT to the ejection time (PAT_ET_) and correlate the findings to CI. The correlation between PAT_ET_ and CI was 0.37 (*p* = 0.11) (supplemental figure 4). The correlation between the product of EF and HR (i.e. EF × HR) and CI was 0.29 (*p* = 0.23) (figure 3).

## 4. Discussion

This study investigated a non-invasive, continuous, time metric between left ventricular depolarization and resulting peak of flow in the PPG, via a waveform derived value called PAT. We found that there is a moderately significant correlative relationship between PAT and invasive CI in pediatric patients after OHT with normal EF. This correlation was slightly enhanced by indexing PAT by HR, by multiplying the average patient HR to the average PAT value. To our knowledge, our study is the first study to compare CI and PAT in a dedicated pediatric population using the gold standard of cardiac catheterization. The lack of correlation between PAT and EF (−0.18, *p* = 0.46) suggests that PAT’s relationship with CI is independent of EF; however, this should be further investigated in a broader range of EF values. Our data suggests that EF does not have a strong correlation with CI with a correlation of 0.02 (*p*-value = 0.91), which is consistent with previously published studies (Schiattarella *et al* 2022, Das *et al* 2023). It is important to note that the patients in our cohort had relatively normal EF after OHT. Additionally, one must recognize that EF, as measured by echocardiography, can have significant variability based on an individual operator (Thavendiranathan *et al* 2013). Given the narrow range of EF values in this cohort, the correlation of EF and PAT should be more thoroughly investigated.

In our data, we saw only a weak correlation between HR and CI, with a Pearson’s correlation of 0.22 (*p*-value = 0.35). However, when we indexed PAT with HR, our correlation improved marginally. CO is defined as the product of HR and Stroke Volume; thus PAT could potentially be a surrogate for stroke volume in pediatric patients. It is important to note that our patient cohort was comprised of patients who underwent OHT, and thus have a relatively denervated heart. Current literature reports that sympathetic reinnervation occurs around 5–6 months, while parasympathetic reinnervation is more delayed, occurring 1–3 years post heart transplant (Awad *et al* 2016). The average time from transplant for our cohort was roughly 5 years; however, the amount of cardiac reinnervation is unknown. The lack of correlation between CI and HR could be potentially due to the dampened innervation of the transplanted heart. Despite the unknown re-innervation of the heart for each individual patient, the product of PAT and HR (i.e. PAT × HR) had the highest correlation to CI.

Previous work has primarily looked at PAT in the context of adult trials as it was known to have a good correlation with stroke volume (Sugo *et al* 2010). An observational study by Fot *et al* (2023), examined a commercial product (esCCO) in 21 adults undergoing elective off pump coronary artery bypass, that utilizes a strategy analogous to PAT. In addition to using non-invasive waveform data, esCCO utilizes a proprietary corrective formula to better predict CO in adults. Furthermore, esCCO requires additional third party software to obtain usable data, where our study only uses the patient’s physiologic data; without corrective formulas. The aforementioned study calibrated their non-invasive measures of CO via transpulmonary thermodilution and found that the agreement between this method of CO monitoring was clinically unacceptable. Additional work by Terada *et al* (2014) evaluated the correlation of esCCO and CO as measured by dye densitography-cardiac output (DDG-CO) in seven pediatric patients. In this study the authors assumed that DDG-CO is analogous to the gold standard for measuring CO, a claim that has not been validated in pediatrics. They found the correlation between esCCO and DDG-CO to be 0.904, which is substantially higher than our presented study of 0.58. However, the study had a relatively small sample size, and did not correct for patient CI, which in pediatrics, is more indicative of optimal oxygen delivery in critically ill children.

Having a continuous, non-invasive modality as a surrogate for CI available at the bedside would greatly impact PICU patients. While having a modest correlation to CI, PAT had a greater correlation than routinely used physiologic metrics such as HR and EF. Additionally, PAT does not require any additional monitors, devices, or proprietary software, making it easily deployed at the bedside and potentially used in any PICU setting. Furthermore, though PAT is far from a perfect correlation, having a multimodality, non-invasive parameter to use and trend at the bedside would be a significant step forward to understanding a patient’s physiology in near real time.

In future studies we hope to show the influence of inotropic medication on PAT, and potentially demonstrate how medications could be accurately titrated to the patient’s physiology in real time under more variable conditions (i.e. Sepsis, Heart Failure, Pediatric Acute Respiratory Failure). PAT is defined as the time of signal propagation from electrical ventricular contraction to signal detection via pulse-oximetry; thus, vascular tone is likely to play a major role in the time of signal propagation and PAT.

This study has several limitations. Our sample size is small and from a single institution; however, similar physiologic studies are of similar, if not smaller, sample size. Additionally, this study examined patients after cardiac transplant, and thus the correlation of PAT and CI in a native heart may be different. Most patients who are admitted to the PICU have dynamic physiology, where intravascular volume, cardiac contractility and cardiac chronotropy are constantly changing. This study looked at relatively healthy children undergoing cardiac catheterization, with normal EF and thus had stable hemodynamics. Most of patients in this study did not have invasive arterial lines, thus waveform analysis of arterial blood pressure tracings, or continuous blood pressure analysis was not able to be done. Perhaps the true association between CI and PAT in pediatric patients will become clearer with a larger sample size and a wider range of hemodynamic values.

Additionally, the degree of cardiac denervation after OHT is unknown in this patient population, so the impact of a chronotropically competent patient on PAT and CI remain an area for investigation before these findings could be generalized to all PICU patients. We hope to explore the relationship of PAT and CI in other pediatric patient populations undergoing cardiac catheterization, such as those with unrepaired ventricular septal defects or atrial septal defects; where chronotropy is expected to be preserved, and accurate CI can be calculated.

The impact of sedation and anesthesia was also not controlled for in this pilot study. The impact of blood pressure, vascular tone, and various anesthetics agents on PAT remain avenues for further exploration within pediatrics.

## 5. Conclusion

Ensuring adequate oxygen delivery and end organ perfusion is crucial in the management of critically ill pediatric patients. Assessing CI at the bedside plays an important role in managing fluid therapy and vasoactive medications. There is a robust amount of non-invasive data that can be routinely captured while patients are admitted in the PICU. Based on our study, PAT derived from routine bedside monitor waveform data, may serve as a valuable, easily deployable, surrogate marker of CI in pediatric patients and deserves further investigation.

## Data Availability

The data cannot be made publicly available upon publication because no suitable repository exists for hosting data in this field of study. The data that support the findings of this study are available upon reasonable request from the authors.
